# Chansu inhibits the expression of cortactin in colon cancer cell lines *in vitro* and *in vivo*

**DOI:** 10.1186/s12906-015-0723-3

**Published:** 2015-07-02

**Authors:** Chun Li, Saeed M. Hashimi, Siyu Cao, Ji Qi, David Good, Wei Duan, Ming Q. Wei

**Affiliations:** Menzies Health Institute, Queensland and School of Medical Science, Griffith University, Gold Coast, 4222 QLD Australia; School of Physiotherapy, Australian Catholic University, Banyo, QLD Australia; School of Medicine, Deakin University, Waurn Ponds, VIC Australia; Molecular and Gene Therapies programme, Menzies Health Institute, Queensland, School of Medical Science, Griffith University, Gold Coast, 4222 QLD Australia

**Keywords:** Apoptosis, Chansu, Cinobufagin, Cortactin and xenograft

## Abstract

**Background:**

Chansu is a transitional Chinese medicine that has been used for centuries as therapy for inflammation, anaesthesia and arrhythmia in China and other Asian countries. Recently, it has also been used for anti-cancer purposes. We have previously shown that Chansu has a huge pro-apoptotic potential on colon cancer cells, but to date the detailed mechanism of this action is not well understood.

**Methods:**

One of the major components of Chansu, Cinobufagin (CBF) was used to treat cancer cells. The expressions of levels of cortactin, an important factor in tumour progression and cancer invasion, were assessed in *in vitro* and *in vivo* experiments. Additional analyses were performed in subcellular protein fractions and immune-fluorescent staining was used to define cortactin protein expression and the changes of location in CBF-treated cells.

**Results:**

CBF strongly inhibited the expression of cortactin in HCT116 cells. There were reductions of both mRNA transcription and protein synthesis, which were more significant in the absence of oxygen *in vitro*. In addition, nuclear translocation of cortactin was observed in HCT116 cells post CBF exposure but not in the negative control, indicating that CBF is likely to interrupt co-localisation of cortactin to cytoskeletal proteins. Most importantly, CBF could diminish the expression of cortactin in human HCT116 xenograft tumours in nude mouse *in vivo*.

**Conclusions:**

CBF inhibits cortactin expression and nuclear translocation in colon cancer cells *in vitro* and in mouse models bearing human colon tumour *in vivo*, suggesting it might disrupt actin-regulated cell movement. Thus, CBF or Chansu could be developed as an effective anti-cancer therapy to stop local invasion and metastasis.

## Background

**Fig. 1:**
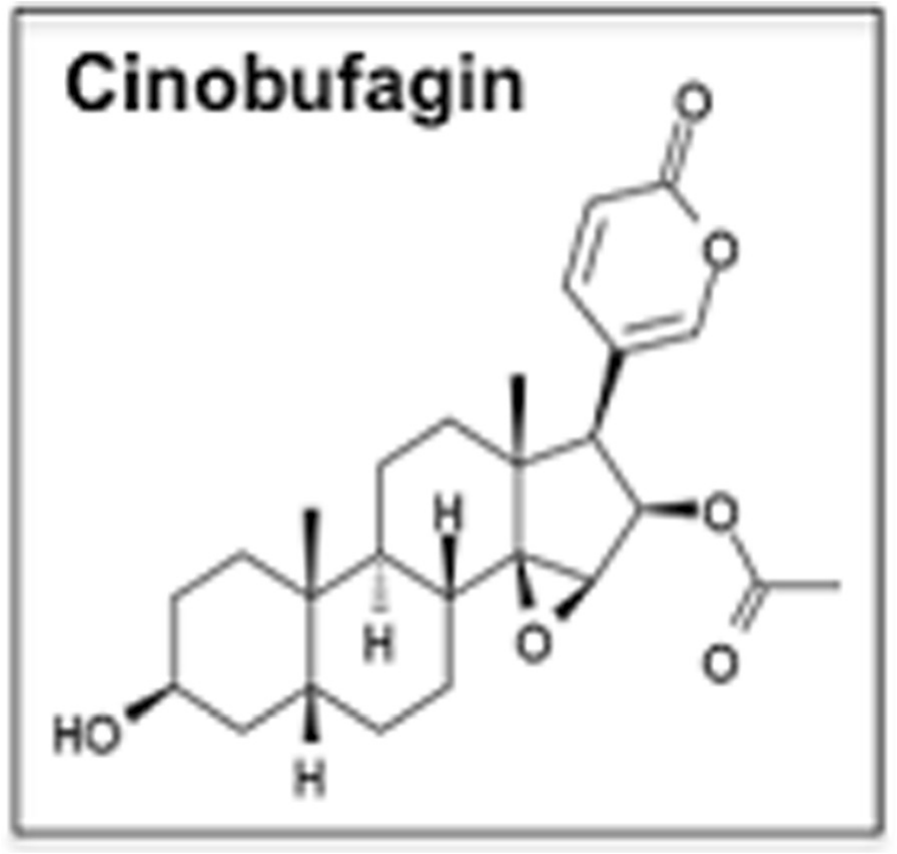
The molecular structure of CBF. CBF possesses a similar function as other typical sodium/potassium-ATPase inhibitors

Chansu is a traditional Chinese medicine extracted from parotoid glands of the Chinese toad (*Bufo gargarizan*). It has been widely used for the treatment of inflammation, anaesthesia and arrhythmia in China, Japan and other Asian countries for centuries [1]. Cinobufagin (CBF) is a major component isolated and purified in the last decade [2]. The compound possesses a digoxin-like structure and is a type of sodium/potassium-ATPase inhibitor (Fig. 1). Recently, other sodium/potassium-ATPase inhibitors have also been reported to impair cancer cell migration through different signalling pathways [3–7].

Cortactin is an important factor involved in cancer cell progression and invasion [8]. It was initially discovered to localise to cortical actin at the cell periphery [9]. During normal cell migration, cortactin is phosphorylated by Src kinase in the C terminal proline-rich domain, while Arp2/3 complex binds to the N terminal of cortactin. The binding of Arp2/3 complex promotes actin polymerisation and facilitates the stabilisation of branched actins [10]. Then the formation of cell motility structures like lamellipodia supports the movement of certain types of cells, including osteoclasts and macrophages [11]. Similarly, phosphorylated cortactin is also able to initiate actin assembly but to form invadopodia in cancer cells, followed by extracellular matrix (ECM) degradation. As a result, detached cells invade surrounding tissues. Thus, cortactin is used as a marker for detection of invadopodia and enrichment of cortactin indicates the metastatic level in a number of cancers [12]. Colon cancer is one of the leading cancer deaths worldwide and most of the patients died from metastatic diseases [13]. Previously, overexpression of cortactin was revealed in a few types of colorectal cancers [14]. Compared with normal tissues, primary cancerous colorectal tissues showed assembly of cortactin in lymph nodes, suggesting a close involvement of cortactin in metastasis of colorectal cancer cells.

In previous work, we demonstrated that CBF induced strong apoptosis in colon cancer cells and a hypoxia-regulated pathway was involved in the drug effect [15]. In the current study, we used CBF as a surrogate marker for Chansu, and further determined the role of CBF in the modulation of cortactin expression in human colon cancer cells. Moreover, we established the effects of CBF treatment under both hypoxic and normoxic conditions, as several of these ion pump inhibitors, including digoxin and ouabain, have been shown to play an anti-cancer role by diminishing hypoxia-inducible factor 1 alpha (HIF-1α) expression [16, 17]. Subsequently, mice bearing HCT116 tumour were also used to reveal that CBF induced a reduction of cortactin synthesis in tumour tissues. Taken together, our findings suggest that the mechanism of Chansu for inhibition of colon cell invasion could be through blocking the interaction between cortactin and actin.

## Methods

### Cell culture and drug treatment

HCT116 and HT29 colon cancer cell lines (provided kindly by Dr Albert Mellick, Griffith University, Australia) were maintained in DMEM (Gibco) supplemented with 10 % FBS (HyClone), 2 mM GlutaMAX (Gibco), 100 U/ml penicillin (Sigma), 100 μg/ml streptomycin (Sigma), 110 mg/L sodium pyruvate (Gibco) and 25 mM HEPES (Gibco), in an atmosphere of 5 % CO_2_ and 95 % air at 37 °C. CBF was purchased from Sigma and dissolved in DMSO (Sigma). 1 μM of CBF solution was applied in all cell line experiments. To achieve a hypoxic condition, cell lines were sealed in GasPak pouches (BD) and placed in an incubator at 37 °C.

### RNA extraction and real-time RT-PCR

In cell lines, total RNA was isolated at different time points using a PureLink RNA mini kit (Ambion), according to the manufacturer’s instructions. For RNA extraction from mouse tumour tissues, fresh tissues (<125 mm^3^) were initially submerged in RNA*later* solution (Ambion) and stored at 4 °C overnight. The subsequent RNA extraction was carried out by following the instructions of TRIzol reagent (Ambion). The concentration of all RNA samples was measured by NanoDrop ND-1000 (Thermo Fisher Scientific). cDNAs of interest were synthesised using SuperScript III RT kit (Invitrogen) according to the manufacturer’s instructions. Real-time PCR was carried out in 20 μl of reaction solution, consisting of 0.4 μM primers (Sigma), 10 μl of Express SYBR GreenER qPCR SuperMixes (Invitrogen) and ddH_2_O. Real-time PCR was performed in iQ5 multicolour real-time PCR detection system (Bio-Rad). The reaction conditions were 50 °C for 2 min and 95 °C for another 2 min, followed by 40 cycles of 95 °C for 15 s, 60 °C for 1 min. Melting curves were monitored by heat-denaturing amplicons over a 35 °C temperature gradient at 0.5 °C/s from 60 to 95 °C. No genomic DNA contamination or pseudogenes were detected. Primers used in real-time PCR were: Human cortactin (Forward: 5′ - AGG TGT CCT CTG CCT ACC AGA A - 3′, Reverse: 5′ - CCT GCT CTT TCT CCT TAG CGA G -3′). Human GAPDH (Forward: 5′ - GTC TCC TCT GAC TTC AAC AGC G - 3′, Reverse: 5′ - ACC ACC CTG TTG CTG TAG CCA A - 3′).

### Western blotting

Cells were scraped in cold PBS and centrifuged down (500 × *g*) to remove methanol. The pellet was resuspended in cold RIPA buffer (Pierce), supplemented with Complete protease inhibitor cocktail tablets (Roche). After centrifugation at 13,000 × *g* for 10 min, the supernatant was collected for further analysis. For protein extraction from mouse tumour tissues, frozen tissues were ground in a mortar and pestle and then immersed in cold RIPA buffer plus protease inhibitor. Further homogenisation was performed by passing the tissues 5–10 times through a 21-gauge needle. After centrifugation at 13,000 × *g* for 10 min, the supernatant was collected and mixed with 1X SDS sample buffer. Protein samples were loaded onto 7 % or 12 % SDS-PAGE gels, running in Mini Trans-Bolt module (Bio-Rad). After gel electrophoresis, proteins were transferred to PVDF membranes (Millipore). The membranes were incubated with primary antibodies against cortactin (1:2000, Abcam) and α-Tubulin loading control (1:5000, Abcam) overnight at 4 °C after a 45 min blocking. Horseradish peroxidise-conjugated goat anti-mouse and anti-rabbit (1:10000, Bio-Rad) secondary antibodies were applied afterwards. SuperSignal chemiluminescent substrate (Pierce) was added to the membranes which were visualised using a VersaDoc MP4000 system (Bio-Rad).

### Subcellular protein extraction

5 × 10^6^ treated HCT116 cells were detached, centrifuged down to form a cell pellet and snap frozen in liquid nitrogen. The subcellular protein extraction followed the manufacturer’s instructions of ProteoExtract Subcellular Proteome Extraction Kit (Calbiochem). Briefly, frozen cell pellets were washed twice in wash buffer and exposed to Extraction Buffer I plus protease inhibitor cocktail. After centrifugation, the supernatant was collected as the cytosolic protein fraction and the pellet was resuspended Extraction Buffer II to isolate the fraction of membrane/organelle proteins. After a series of centrifugation and usage of specific extraction buffer III ~ IV, the nuclear and the cytoskeletal matrix protein fractions were separated.

### Immunocytochemistry

Sterile coverslips were placed in a 24-well plate and cancer cells were seeded at a density of 1.5 × 10^5^ cells per well. After overnight incubation at 37 °C, cells were adherent to the coverslips. Treated cells were washed in ice cold PBS and fixed in 100 % methanol at −20 °C for 10 min, followed by washing in ice cold PBS twice, shaking gently. Usage of 0.2 % Triton X-100 (Sigma) in PBS to permeabilise samples was for no more than 10 min, followed by 3 times of wash in PBST. Cells were then blocked in block buffer (3 % normal goat serum and 0.5 % BSA in 0.01 M PBS) for 30 min and sequentially incubated with primary antibody cortactin (1:300, Abcam) overnight at 4 °C. The goat anti-rabbit Texas Red (1:1500, Abcam) secondary antibody was applied in dark for 1 h, followed a counter DAPI staining (Molecular Probes). All the coverslips were sealed onto microscope slides using ProLong Gold antifade reagent (Molecular Probes) and kept in dark for 24 h. Fluorescence images were visualised using confocal microscope FV1000 (Olympus).

### Xenograft establishment and CBF treatment

To establish nude mouse models bearing HCT116 tumour, 5 × 10^6^ HCT116 cells in PBS (200 μl) were subcutaneously injected into each mouse using 30 - G needles. After tumour growth for 2 weeks, 14 female BALB/c nude mice (aged 8 weeks and weighing 16–18 g) were equally divided into two groups (7 mice per group, *N* = 7): intraperitoneal (i.p.) injection and control, with average tumour size of each group of about 0.32 cm^3^. CBF was dissolved firstly in absolute ethanol and diluted in 10 % propylene glycol solution. To prepare 50 ml injection solution, 10 mg of CBF was dissolved in 4 ml of absolute ethanol and diluted in 10 % propylene glycol solution to reach 0.2 mg/ml. The daily dose given to i.p. group was 1.5 mg/kg, while an equal amount of injection solution without CBF was given as a control. Mice were sacrificed when the tumour grew to 1 cm^3^. Tumour tissue specimens were taken for subsequent RNA and protein analyses. All experiments involving animals were approved by Griffith University (AEC No. MSC/01/08).

## Results

### CBF inhibits mRNA and protein expression of cortactin in HCT116 cells

**Fig. 2:**
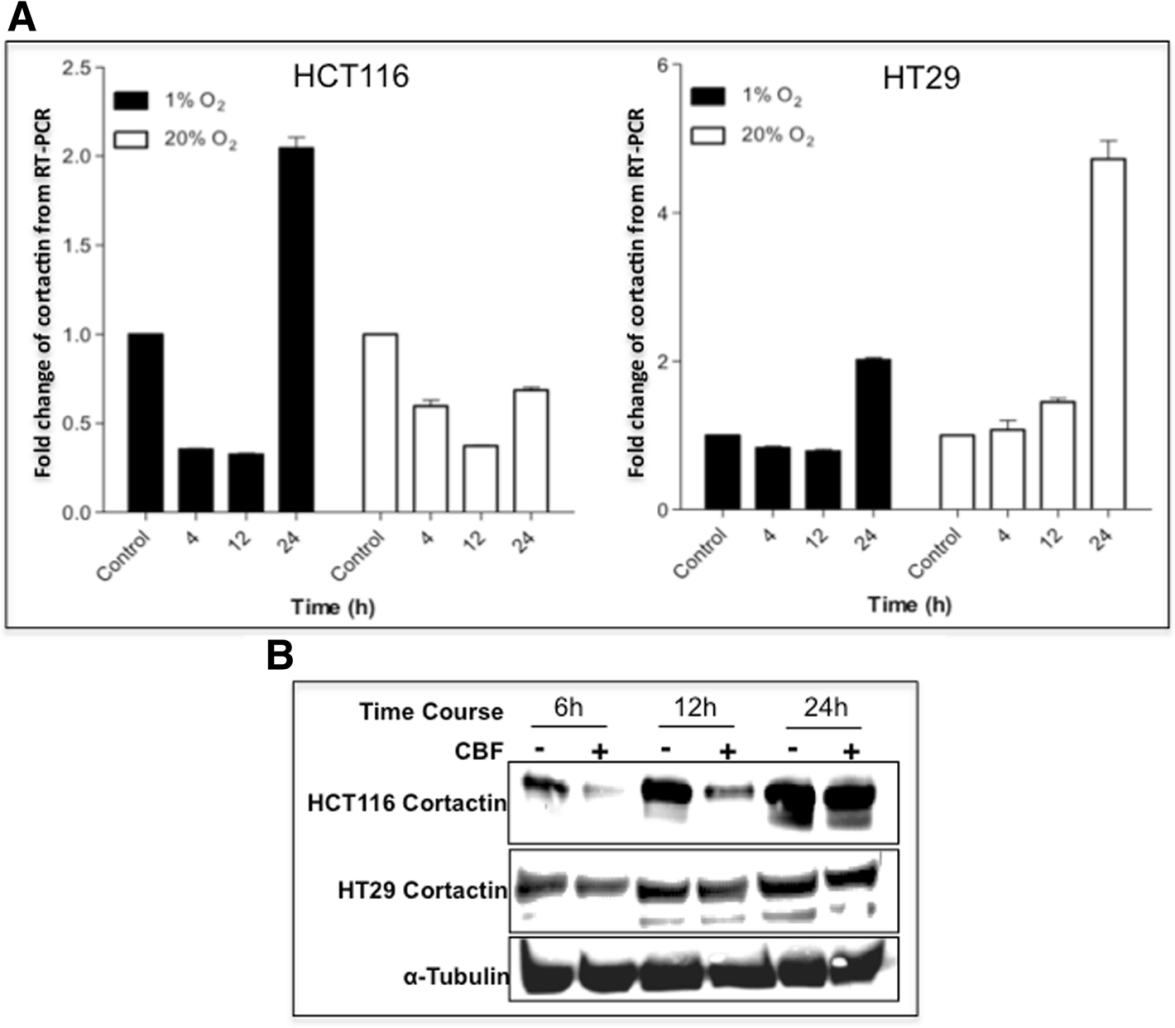
CBF affected cortactin mRNA and protein expression in human colon cancer cell lines HCT116 and HT29. **a** The mRNA transcription of cortactin in CBF-treated HCT116 and HT29 cells. 1 μM of CBF was added to HCT116 and HT29 cells which then incubated under hypoxic and normoxic conditions. The cells were collected at different time points and analysed. Results are means with standard errors from four replicates. The level of GAPDH as a control was set to 1.0. **b** Cortactin protein inhibition only in HCT116 cells (1 % oxygen). The expressions of cortactin were significantly inhibited by CBF in HCT116 cells at 6 h and 12 h, but undetectable in HT29 cells

Cortactin is overexpressed in colon cancer tissues [18] and has been shown to be an important factor in tumour progression and cancer invasion. In this study, we treated colon cancer cells (HCT116 and HT29) with CBF (1 μM). In order to mimic the colon cancer microenvironment, the colon cancer cells were also exposed to hypoxic and normoxic conditions, respectively (Fig. 2a). Our results showed that there was a decreased expression of cortactin mRNA in HCT116 cells under 1 % oxygen. Such a decrease was swift, but only lasted less than 24 h, followed by a significant increase of cortactin transcription. As the level of oxygen increased to 20 %, the transcription of cortactin, however, exhibited an overall reduction in HCT116 cells within 24 h. On the other hand, cortactin mRNA level in HT29 cells only slightly altered during the initial 12 h under different oxygen conditions. Subsequently, a sharp elevation of mRNA level was observed at 24 h with 2 folds increase in hypoxia and almost 5 folds increase in normoxia.

The expression of cortactin protein under hypoxic conditions was consistent with mRNA message (Fig. 2b). The inhibition of cortactin at 6 and 12 h in HCT116 cells was significant, but not at 24 h. This inhibitory role was barely detected in CBF-treated HT29 cells at any time points. Taken together, our finding showed that CBF inhibits mRNA and protein expressions of cortactin in HCT116 cells and this inhibition is swift but not sustainable. In fact, all the increases of cortactin mRNA at 24 h time point implied an interference between CBF-induced inhibition and cortactin transcription. Such elevated mRNA levels might also result in no down-regulation of protein expression at 24 h.

### CBF affects distribution of cortactin in HCT116 cells under hypoxic conditions

**Fig. 3:**
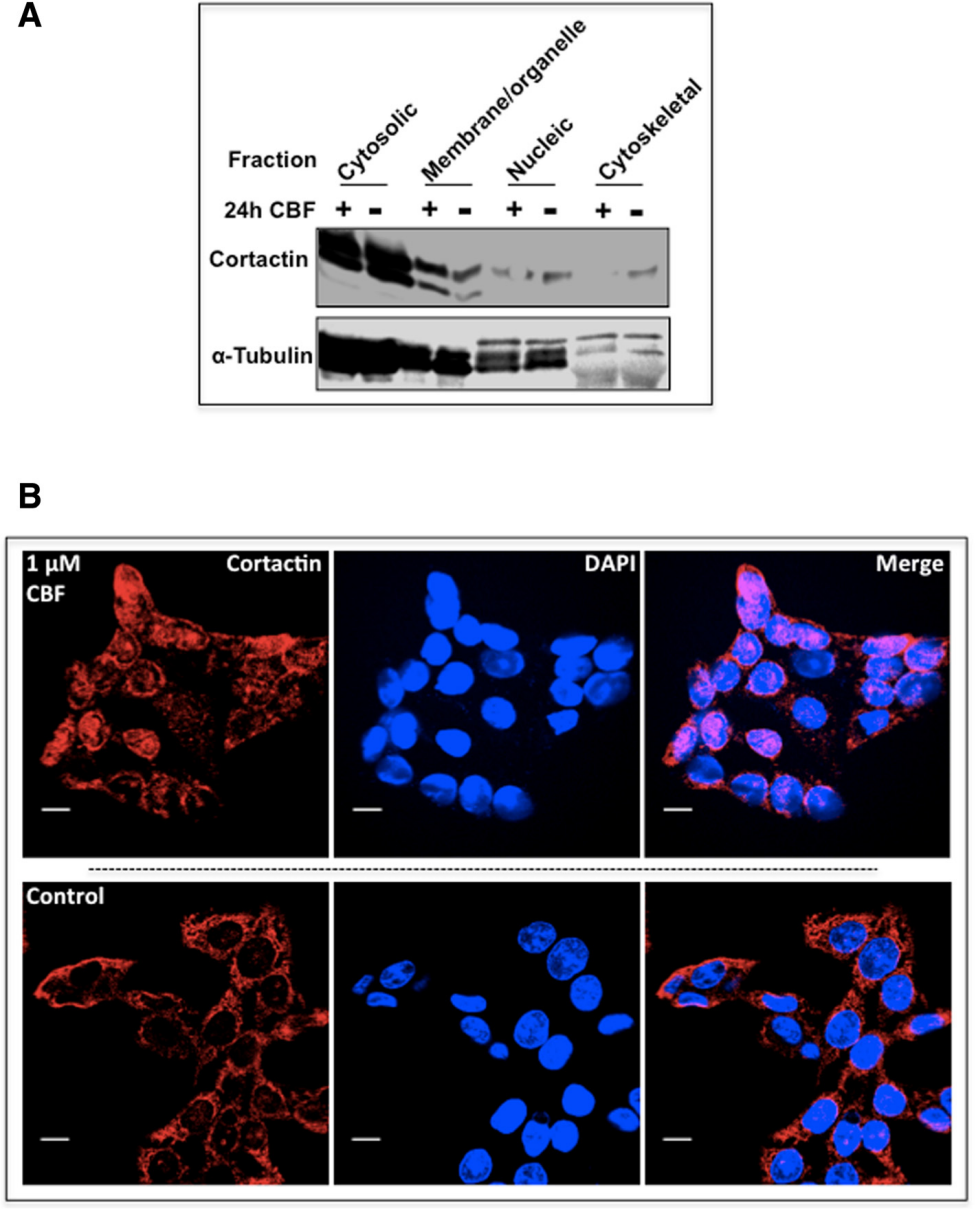
CBF changed the distribution of cortactin in HCT116 cells (1 % oxygen). **a** Subcellular protein extraction of four fractions, which are cytosolic, membrane/organelle, nucleic and cytoskeletal fractions. Cortactin expression was diminished significantly in the fraction of cytoskeletal proteins after exposure to 1 μM of CBF. **b** Co-localisation of cortactin in treated HCT116 cells. Under a hypoxic condition, HCT116 cells were incubated with 1 μM CBF for 24 h. The subsequent staining revealed a colour overlapping, indicating that CBF induced a nuclear translocation of cortactin. Scale bars equal 10 μm

To further investigate CBF-induced cortactin inhibition, we examined the subcellular protein expression in HCT116 cells in hypoxia. Subcellular protein fractions indicated that the level of cortactin in cytoskeletal fraction was diminished after CBF treatment, suggesting the dissociation of cortactin and cytoskeletal proteins (Fig. 3a). Generally, cortactin is localised in the cell periphery. However, immunostaining showed that CBF exposure led cortactin to shift towards the nucleus (Fig. 3b).

### CBF inhibits mRNA and protein expression of cortactin in nude mouse models bearing HCT116 tumour

**Fig. 4:**
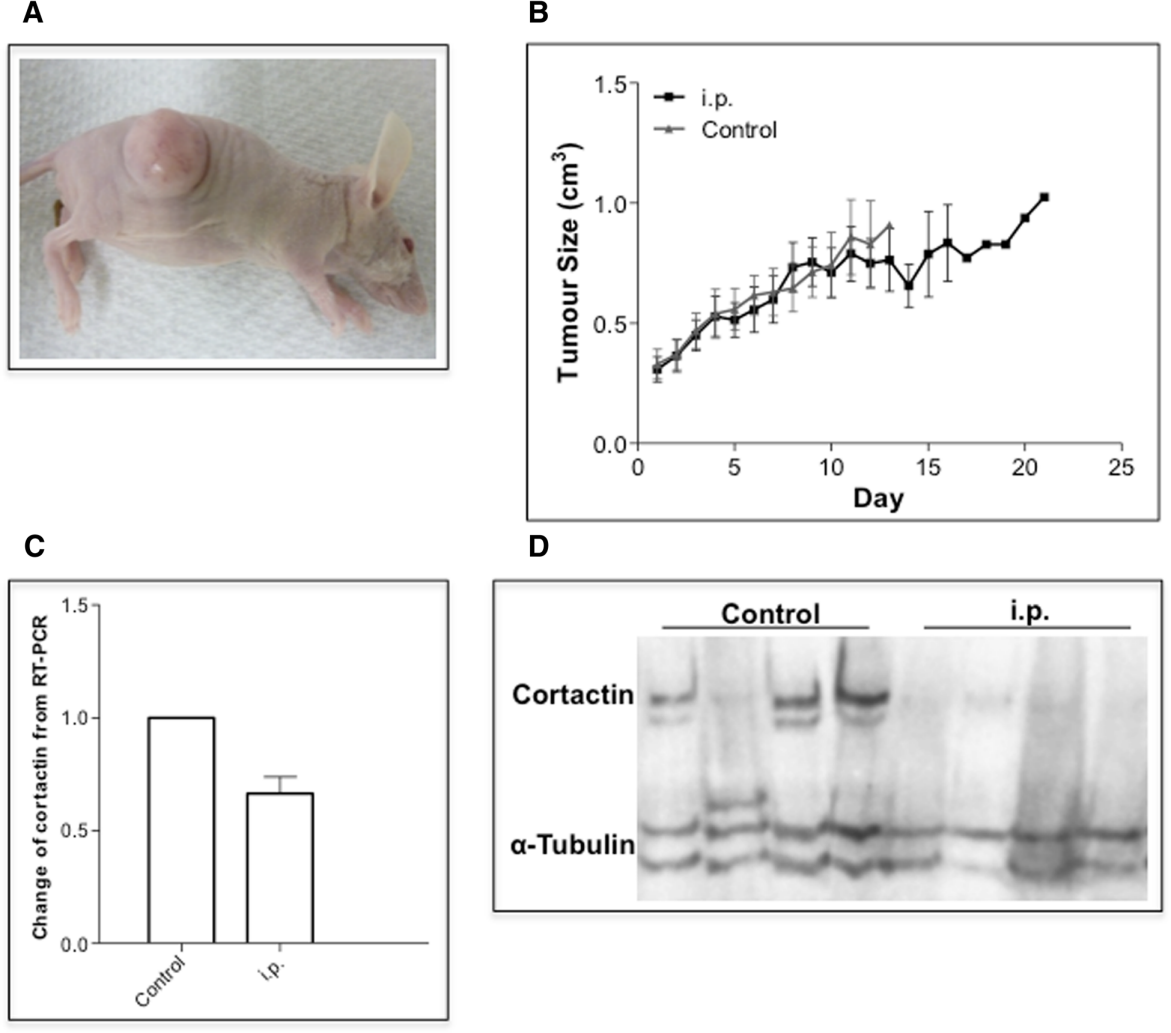
CBF inhibited cortactin synthesis in xenografts. **a** A nude mouse model bearing HCT116 tumour. The mouse was sacrificed when the tumour size reached 1 cm^3^. **b** Tumour size vs days of treatment (N = 7). Compared with the control, the i.p. group showed a slight suppression of tumour growth after the drug injection. **c** Analysis of cortactin mRNA level in tumour tissues. A significant decrease of cortactin mRNA level presented in i.p. group. Results are means with standard errors from four replicates. The level of GAPDH as a control was set to 1.0. **d** Abundant cortactin protein expression only in the control group. The i.p. group showed diminished expression of cortactin. The absence of cortactin in lane 6 of control group was an exemption with an unknown reason

HCT116 cells were implanted in lateral right back of nude mice (Fig. 4a). When the average of tumour size reached 0.32 cm^3^, the CBF treatment started by i.p. injection. The mice were sacrificed once the tumour grew to 1 cm^3^ and tumour tissues were collected. Between two groups, the average of tumour size was found to be slightly suppressed by CBF in i.p. group (Fig. 4b). Moreover, cortactin mRNA level in i.p. group was significantly reduced (Fig. 4c). The RT-PCR was conducted using mouse tumour tissue samples from different groups. Interestingly, the protein inhibition was found in the treatment group (Fig. 4d), demonstrating that CBF repressed cortactin synthesis in xenografts.

## Discussion

Recently, Chansu is being used as an anticancer agent. Clinical use has shown that it improved the quality of life in patients with advanced gallbladder cancer [19]. As a major component in Chansu, CBF was used as surrogate marker to study the mechanism of anticancer activity of Chansu. We previously demonstrated that CBF (1 μM) induced effective apoptosis in colon cancer cell lines HCT116 and HT29, with 57 % and 30.5 % of cell death, respectively [15]. We also showed that a key mediator of cell motility and metastasis, cortactin, was significantly inhibited by CBF in HCT116 cell line and in xenografts HCT116 tumours.

Although the overexpression of cortactin in HCT116 cells was identified in previous studies [14, 20], this report also showed that there is a high level of cortactin in colon cancer cell line HT29 as well. The inhibition of transcription and protein levels of cortactin in HCT116 cells was observed in HCT116 but not in HT29 cells, reflecting that CBF appears to induce strong suppression in HCT116 cells but has limited efficacy on HT29 cells. Therefore, CBF is unlikely to strongly target cortactin in all colon cancer cells. On the other hand, the inhibition occurred swiftly but was overcome within 24 h. As the half-life of cortactin protein is about 8.9 h [21], CBF seems to have a fast on-off effect on the protein. A sharp increase of cortactin mRNA under hypoxic conditions at 24 h revealed a possibility that the cancer cells in a hypoxic tumour microenvironment appear to be able to resistant to CBF. Moreover, the 24 h increase of cortactin mRNA was detected in both cell lines regardless to the oxygen levels, suggesting that it could be resulted from the up-regulation of mitogen-activated protein kinase (MAPK) pathways [22, 23]. Drugs like ouabain and digoxin have been shown to be capable of activating MAPK by binding to sodium/potassium-ATPases, resulting in the release of Src kinase [22, 23]. As cortactin is a substrate for Src [9], the transcription level of cortactin could be elevated, followed by abundant expression of cortactin protein under our assay conditions.

Apart from the *in vitro* experiments in 1 % oxygen, the suppression of cortactin was also shown in tissue samples of nude mice bearing HCT116 tumours. The results confirmed the inhibitory role of CBF in xenografts *in vivo*. Although cortactin inhibition did not sustain in HCT116 cells within 24 h, CBF is still able to block cortactin synthesis in HCT116 tumour tissues of mouse models. We hypothesized that this could be due to the down-regulation of nuclear factor kappa beta (NF-κB), as Hill *et al.* elucidated that inhibition of p65 Rel A subunit of NF-κB by IκKinase-2 led to a significant suppression of cortactin mRNA transcription in breast cancer MCF7F-B5 cells [24]. Our previous results from multi-pathway arrays revealed that CBF significantly impedes NF-κB activity in HCT116 cells [15]. Thus, the long-term inhibition of cortactin *in vivo* could be a consequence of CBF-induced deactivation of NF-κB. Taken together, our data establishes the inhibitory role of CBF in cortactin synthesis in a HCT116 cells.

In addition to the general down-regulation of cortactin protein, the CBF treatment also altered the distribution of cortactin in HCT116 cells under hypoxic conditions. The nuclear translocation of cortactin in cancer cells was a new observation. Here, the cause for this shift is still unclear. Oddly, the total level of cortactin in nucleus did not boost after the drug exposure, which left a question whether cortactin was only dragged to the nucleic surface and then degraded swiftly. The pioneer work of Hering and Sheng (2003) showed a nuclear accumulation of cortactin mutant in dendritic spine morphogenesis [25]. They discovered that there is a tandem repeat region within the N-terminal half of cortactin and the deletion of this region targets cortactin to the nucleus of dendritic cells rather than dendritic spines. Thus, one possible explanation for the nuclear import of cortactin in CBF treated cells is the interruption of the tandem repeats. Nevertheless, the nuclear translocation of cortactin was not detected in HT29 cells or the tissue samples from mouse models bearing HCT116 tumours. Therefore, more investigations of the nuclear import need to be done in the future.

## Conclusions

In conclusion, CBF possess the unique capacity to inhibit the overexpression of cortactin in HCT116 cells and nude mouse models bearing HCT116 tumours. It seems likely that this is the mechanism for Chansu to be used as an anticancer agent, inhibiting colon cancer cell proliferation and metastasis.

## Abbreviations

CBF: Cinobufagin
HIF-1α: Hypoxia-inducible factor 1 alpha
ECM: Extracellular matrix
i.p.: Intraperitoneal
MAPK: Mitogen-activated protein kinase
NF-κB: Nuclear factor kappa beta
